# Performance Analysis of Air Gap Membrane Distillation Process Enhanced with Air Injection for Water Desalination

**DOI:** 10.3390/membranes14110232

**Published:** 2024-11-06

**Authors:** Jonathan Ibarra-Bahena, Ulises Dehesa-Carrasco, Rogelio Servando Villalobos-Hernández, Sofía Garrido-Hoyos, Wilfrido Rivera

**Affiliations:** 1 Instituto de Energías Renovables, Universidad Nacional Autónoma de México, Privada Xochicalco S/N, Temixco 62580, Mexico; jibarra@ier.unam.mx; 2 Instituto Mexicano de Tecnología del Agua, Paseo Cuauhnáhuac 8532, Jiutepec 62550, Mexico; ulises_dehesa@tlaloc.imta.mx (U.D.-C.); sgarrido@tlaloc.imta.mx (S.G.-H.); 3 Centro Nacional de Referencia en Parasitología Animal y Tecnología Analítica, Carretera Federal Cuernavaca-Cuautla 8534, Jiutepec 62550, Mexico; rogelio.villalobos@senasica.gob.mx

**Keywords:** desalination, AGMD, air injection, gas-liquid two-phase flow

## Abstract

Water scarcity is a global issue, and desalination is an alternative to providing fresh water. Renewable energies could be used in thermal desalination to produce freshwater from high saline concentration solutions. In this paper, the experimental performance of an air-injection-Air Gap Membrane Distillation (AGMD) module is presented. The effect of the operation parameters (saline solution temperature, air flow, and salt concentration) on the distilled water rate was evaluated. The air injection enhanced the distilled water rate by 22% at the highest air flow and a solution flow rate of 80 °C, compared to the conventional condition (without air injection) at a salt concentration of 100,000 ppm. Under the same operating conditions, the increase was 17% at a salt concentration of 70,000 ppm. The maximum distilled water rate was 14.10 L/m^2^·h at 80 °C and an airflow of 1.5 L/min with the highest salt concentration, while it was also 14.10 L/m^2^·h at the lower salt concentration was 14.10 L/m^2^·h. The distilled water quality also improved as the air flow increased, since a conductivity reduction of 66% was observed. With the described mathematical model, 94% of the calculated values fell within ±10% of the experimental data for both salt concentration conditions.

## 1. Introduction

Climate change, population growth, and economic activities cause water scarcity in many regions of the world. Of all the water on Earth, around only 3% is suitable for human consumption in agriculture, industry, urban, and domestic sectors [1]; the remainder is in the oceans as saline water. In this regard, the desalination process is an alternative to freshwater production. In the last decades, thermal desalination using Multistage Flash Distillation (MSF) and Multi-Effect Distillation (MED) were the most-used technologies, accounting for 84% of the global desalination capacity; however, this decreased to 25% in 2019, while Reverse Osmosis (RO) increased by up to 75% at the same period time [2]. Despite thermal desalination being a suitable option for feedwater sources with high salinity concentration, the energy requirement (thermal and electrical) is the main drawback; however, by coupling thermal renewable energy sources, a positive environmental impact can be produced [3]. In conventional thermal desalination processes, such as MSF, MED, or thermal vapor compression, the saline feedwater is boiled to separate the freshwater. However, other emerging desalination processes, such as Humidification–Dehumidification (HDH), Membrane Distillation (MD), and others, do not require reaching the boiling point of the saline feedwater. In addition, these processes have other advantages: applied pressure is not required; high rejection of non-volatile components; lower operating temperatures; corrosion is avoided since polymeric materials are used; high concentration factor which is suitable for Zero Liquid Discharge (ZLD) solutions; simple operation and maintenance; and they are ideal for small-scale application in remote zones [4,5,6].

MD is a low-temperature separation process. The driving force is the vapor pressure difference on both sides of a hydrophobic membrane caused by the temperature gradient generated across the membrane surface. Due to the features of MD (low operating temperature and pressure, near-total salt rejection, modular system design, and low-enthalpy sources such as renewable energy sources), it has the potential to address the disadvantages of the conventional RO process [7]. However, there are some problems limiting the performance of MD: membrane wetting, membrane fouling, and temperature polarization (TP). The latter is related to the driving force loss by thermal gradients in the fluids, which reduced the mass transfer across the membrane and, consequently, the freshwater production [8]. In this regard, the permeate flux can be enhanced when the flow characteristics are improved by increasing the flow rate or turbulence generation [9]. The first method is to increase the solution flow rate, which enhances the convective heat transfer through the boundary layer; therefore, the difference in vapor pressure (driving force) increases, thus increasing freshwater production. However, the energy required for solution pumping increases, and economic feasibility could be affected; additionally, the membrane durability could be reduced [10,11]. The second method is gas injection, producing a two-phase flow regime. Several authors have studied this. Dong et al. [12] reported a novel slug flow-enhanced Vacuum Membrane Distillation (VMD) system. According to the authors, the permeate flux increased by 28.1% with the proposed system concerning the conventional VMD process. In addition, an antifouling effect was observed since, after a fouling test, the permeate flux was reduced by 2.2% while the conventional VMD was reduced by 15.2%. Kim et al. [13] carried out an experimental and theoretical study on the effect of microbubble two-phase flow on the performance of direct-contact MD. A swirl-flow-type microbubble generator was used to produce a two-phase flow regime. The permeate flux was 18% enhanced at 40 °C of solution temperature and with an air flow rate of 50 cc/min. In another paper, Dong et al. [14] described a Two-Phase flow-enhanced Direct Contact Membrane Distillation (TP-DCMD) system and analyzed the effect of a two-phase flow regime on the heat and mass transfer processes and the membrane fouling. The authors pointed out that the permeate flux was improved by 27%, and the fouling effect reduced this parameter by only 6.6%. In contrast, in the conventional DCMD process, the permeate flux decreased by 38.2% due to the fouling effect. Cao et al. [15] proposed a Bubbling and Vacuum-enhanced Direct Contact Membrane Distillation (BVDCMD) for seawater desalination. The authors carried out a numerical simulation and tested four gases (O_2_, air, N_2_, and H_2_) and concluded that the permeate flux using H_2_ was 144% higher than the conventional DCMD process due to the lower viscosity of this gas. Kim et al. [16] proposed an air-assisted swirling flow-type microbubble generator to improve the permeation flux of a DCMD module. According to the authors, the airflow rate effect on DCMD permeation flux was relevant, and it increased by 37% with an airflow rate of 50 cc/min, with respect to the conventional DCMD. Wibisono et al. [17] reported an extensive review regarding the two-phase flow in the MD process.

Among the MD configurations, the AGMD is the most versatile, and it can integrate into several processes that involve compound separation or component recovery [18,19]. However, in the AGMD, the stagnant air layer provides an additional mass transfer resistance, which reduces the permeate flux. In this regard, several improvements to enhance freshwater production have been proposed, such as membrane materials [20,21], multistage configurations [22,23], vortex generation in the fluid channels [24,25], heating and cooling improvement surfaces [26,27,28], and non-stagnant air gaps [29,30]. As was described previously, gas injection generates hydrodynamic instabilities in the feed channel, reducing the membrane fouling and the temperature polarization. Additionally, it can increase the transmembrane pressure difference, increasing freshwater production [17]. However, the literature about the gas injection with the AGMD configuration is scarce: Pan et al. [31] analyzed the gas–liquid two-phase flow in an AGMD module for water desalination with nitrogen as the injection gas. According to the reported results, the gas injection increased the freshwater permeate flux by 4.7 times; in addition to this, the concentration polarization coefficient was smaller while the mass transfer coefficient increased with gas injection than without it.

Based on the literature review, gas injection improves the performance of the MD modules; nevertheless, the effect on the AGMD configuration has been poorly studied, especially at high saline concentrations. Therefore, the present paper presents a novel air-injection-AGMD system for brine treatment. The impact of the operation parameters on the freshwater rate was analyzed. The aim of this research is to demonstrate the technical feasibility and performance of the proposed system for the desalination process with brine concentration from 70,000 to 100,000 ppm. In addition, a heat and mass transfer model is presented which could improve future new AGMD design.

## 2. Methodology

### 2.1. Desalination Process Description

**Figure 1:**
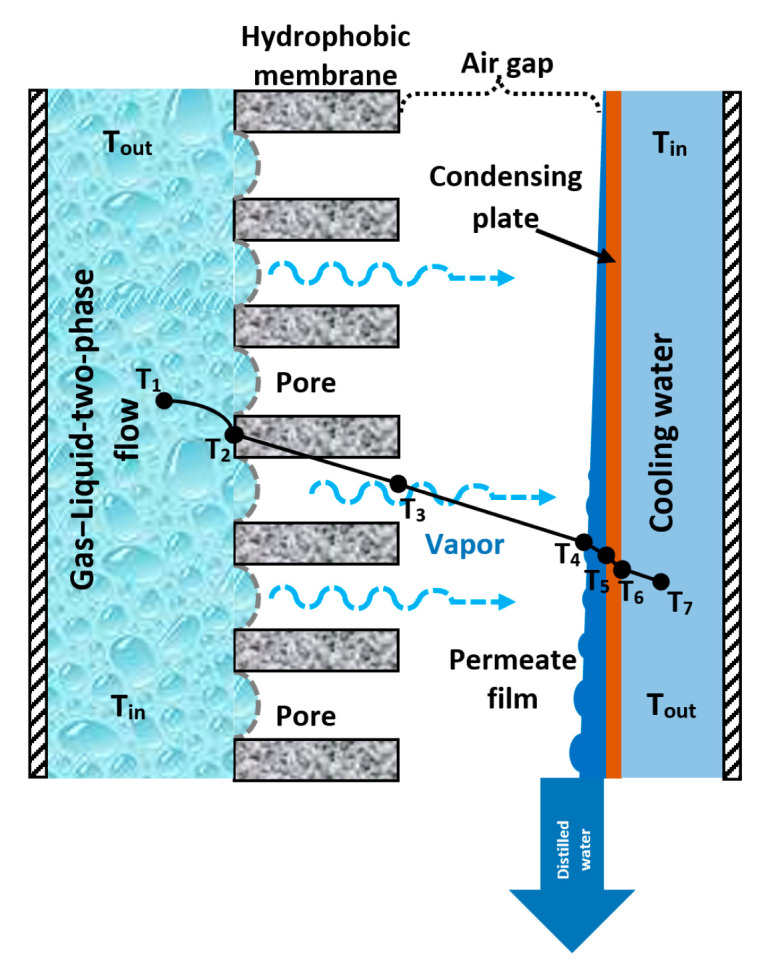
Experimental configuration.

The proposed device integrates the AGMD and the air injection, and it operates as follows: the hot feed stream (saline water) is mixed with a pressurized air stream to generate microbubbles and produce a gas–liquid two-phase flow, which is in contact with a hydrophobic membrane to evaporate a part of the water at the liquid–membrane interphase. The water vapor crosses the membrane and the air gap to the cooling surface (cooling plate), where it is condensed, and finally, the distilled water flows out of the device. Figure 1 shows a schematic diagram of the experimental configuration.

### 2.2. Experimental Setup

**Figure 2:**
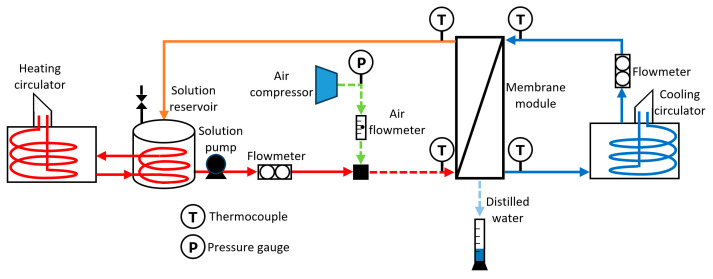
Schematic diagram of the experimental setup.

The experimental setup included a membrane device, a heating system, a cooling source, an air compressor, a solution reservoir, a solution pump, and the instruments to measure the operation parameters, as shown in Figure 2. A heating circulator integrated with the heating system included temperature and flow controls, along with a stainless-steel helical coil heat exchanger to heat a 30 L solution reservoir. On the other hand, the cooling source was a cooling circulator with temperature control and an integrated pump. A 2.5 hp electrical air compressor injects air into the solution before entering the membrane module. The volumetric airflow (*V_G_*) was measured with an analogical flowmeter. The distilled water was measured with a volumetric graduate test tube. Type T thermocouples were placed at the input and output ports of the membrane module. A multiparameter device (from Hanna Instruments, Smithfield, RI, USA) was used to measure the distilled water conductivity. A data acquisition unit, Agilent, and the Agilent HP Vee Pro software were used to record the operating temperatures and volumetric flows. The uncertainties of the measured variables and instruments used in the experimental test runs are shown in Table 1. Two salt solutions with 98% NaCl and 2% CaSO_4_·2H_2_O were prepared with deionized water at 70,000 ppm and 100,000 ppm of salt concentration. These concentrations and chemical elements are in the rejected brine produced by the RO systems [32]. The operation of the system is as follows: the feed solution is preheated with the heating circulator to reach the operating temperature. After that, the feed solution is pumped through the membrane module, and the flow is adjusted and measured with the liquid flow meter. The pressure air flow is measured with the air flow meter and is mixed with the feed solution. The system runs continuously for 30 min; after that, the distilled water is collected and measured with a volumetric graduate test tube. This procedure is repeated for each solution temperature, air flow, and salt concentration.

**Figure 3:**
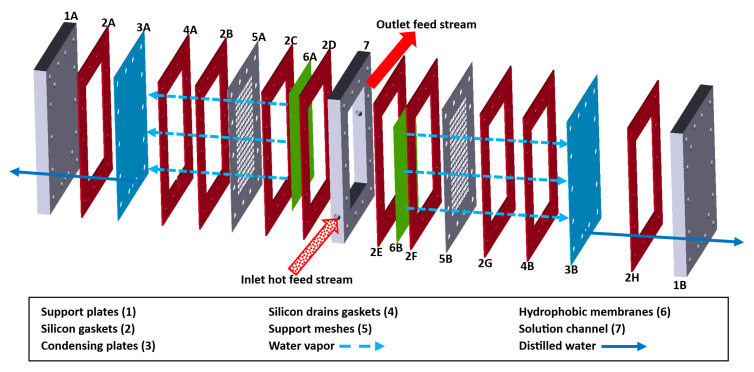
Experimental membrane device configuration.

The membrane module was integrated using three polymeric plates (two support plates and the solution channel) with 310 mm length, 220 mm wide, and 25.4 mm thickness; thermal resistant silicon gaskets with 3 mm thickness; two metallic meshes to avoid the membrane deforming due to the solution flow; two aluminum cooling plates with 0.4 mm thickness and two PTFE hydrophobic membranes with 0.22 µm of pore size (*d_p_*), porosity (*ε*) of 75%, and thickness (*δ_mem_*) of 300 µm. The effective membrane area was 240 cm^2^. 28 bolts and nuts were used to hold the device. Figure 3 shows a schematic diagram of the experimental membrane module. The experimental operating conditions are shown in Table 2.

## 3. Heat and Mass Transfer Model

A two-phase flow heat and mass transfer model was used to estimate the global parameters of the system considering the following assumptions:

(1) The membrane unit operates at steady state conditions.
(2) Thermophysical properties are constant.
(3) Gas and liquid phases are assumed to be incompressible fluids.
(4) The heat and mass transfer processes occur in one dimension.
(5) Gas and liquid phases are fully mixed.
(6) Liquid–vapor equilibrium exists at the evaporation and condensation interphases.

The energy balance of the membrane unit can be assumed as follows:

(1) $$Q_{in}=Q_{f}+Q_{out}$$

*Q_in_* is the inlet heat load [kJ/s]

(2) $$Q_{in}={\dot{m}}_{L}H_{L,in}+{\dot{m}}_{G}H_{G,in}$$

where *ṁ* is the mass flow rate [kg/s] and *H* is the enthalpy [kJ/kg]. The subscripts *L* and *G* mean liquid phase and gas phase, respectively.

*Q_out_* is the outlet heat load

(3) $$Q_{out}=\left({\dot{m}}_{L}-N_{w}A_{m}\right)H_{L,out}+{\dot{m}}_{G}H_{G,out}$$

where *A_m_* is the membrane area [m^2^] and *N_w_* is the distilled water flux [kg/m^2^·h].

*Q_f_* is the heat load transferred from the feed fluid to the membrane:

(4) $$Q_{f}=A_{m}h_{1}\left(T_{1}-T_{2}\right)$$

where *h* is the convective heat transfer coefficient [W/m^2^·°C], *T*_1_ and *T*_2_ are the salt solution temperature and membrane wall temperature at the feed side, respectively [°C], according to the Figure 1.

In addition, *Q_f_* can be expressed as the sum of the heat load required for the water evaporation and the heat load transferred to the membrane by conduction, thus:

(5) $$Q_{f}=A_{m}h_{1}\left(T_{1}-T_{2}\right)=N_{w}{A_{m}\lambda }_{w}+\frac{k_{m}A_{m}}{\delta_{m}}\left(T_{2}-T_{3}\right)$$

where *λ_w_* is the latent heat of vaporization of water [kJ/kg], *k* is the thermal conductivity [W/m·°C], and *δ* is the thickness [m]. *T*_3_ is the membrane temperature at the gap side. The subscript *m* means membrane.

The correlation proposed by Groothuis and Hendal [33] for a two-phase flow regime was used to calculate the Nusselt number, which is related to *h*_1_:

(6) $$Nu=a{Re}_{GL}^{b}{Pr}_{L}^{\frac{1}{3}}{\left(\frac{\mu_{1}}{\mu_{2}}\right)}^{0.14}$$

(7) $${Re}_{GL}=\frac{d_{h,G}v_{G}\rho_{G}}{\mu_{G}}+\frac{d_{h,L}v_{L}\rho_{L}}{\mu_{L}}$$

(8) $${Pr}_{L}=\frac{\mu_{L}C_{p,L}}{k_{L}}\;\;$$

where *Re* is the Reynolds number, *Pr* is the Prandtl number, *µ* is the viscosity [Pa·s], *d_h_* is the equivalent hydraulic diameter [m], *v* is the velocity [m/s], *ρ* is the density [kg/m^3^], *Cp* is the specific heat capacity [kJ/kg·°C], and *k* is the thermal conductivity. The empirical coefficients *a* and *b* were taken from the reported literature and are dimensionless [31]. The subscripts *GL*, *G*, and *L* mean two-phase flow, gas phase, and liquid phase, respectively.

Substituting Equations (2), (3) and (5) into Equation (1), the water distilled flux (*N_w_*) is calculated:

(9) $$N_{w}=\frac{{\dot{m}}_{L}\left(H_{L,in}-H_{L,out}\right)+{\dot{m}}_{G}\left(H_{G,in}-H_{G,out}\right)-\frac{k_{m}A_{m}}{\delta_{m}}\left(T_{2}-T_{3}\right)}{A_{m}\left(\lambda_{w}-H_{L,out}\right)}$$

When *ṁ_G_* = 0, the driving force of the mass transfer is the partial pressure difference between the evaporation interphase (*p*_1_–*p*_3_). However, the mass transfer flux is limited by the global mass transfer coefficient (*K_ov_*):

(10) $$N_{w}=K_{ov}\left(p_{2}-p_{4}\right)$$

The global mass transfer coefficient [kg/m^2^·h·kPa] involves the Knudsen diffusivity, the molecular diffusivity, and the mass transport of water vapor across the air gap [34] and can be expressed as:

(11) $$\frac{1}{K_{ov}}=\frac{1}{K_{m}}+\frac{1}{K_{gap}}$$

where:

(12) Km=MwRTmemδmem23rpετ8RTmemπMw12−1+DvapτPplm−1−1

(13) $$K_{gap}=\frac{M_{w}}{RT_{gap}}\frac{D_{vap}}{\delta_{gap}}\frac{P}{p_{ml}}$$

The first term at the left in Equation (12) is related to the Knudsen diffusivity, and the second is the molecular diffusivity. *M_w_* is the water molecular weight [kg/kmol], *R* is the universal gas constant [kPa·m^3^/kmol·K], *P* and *p_lm_* are the total operating pressure and the logarithmic-mean pressure [kPa], respectively, and *δ_mem_* [m], *ε*, and *τ* are the thickness, porosity, and tortuosity of the membrane, respectively. The last two terms (porosity and tortuosity) are dimensionless. *D_vap_* is the water vapor/air binary mass diffusion coefficient [m^2^/s], and *r_p_* is the membrane’s mean pore radius [m]. The internal temperatures were calculated according to mathematical models reported in the literature for AGMD [35,36,37].

## 4. Results

### 4.1. Distilled Water Rate

**Figure 4:**
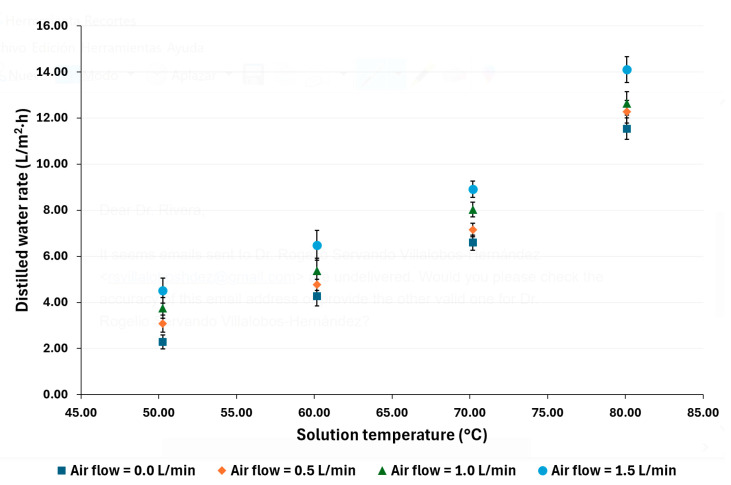
Experimental distilled water rate for a salt concentration of 100,000 ppm.

**Figure 5:**
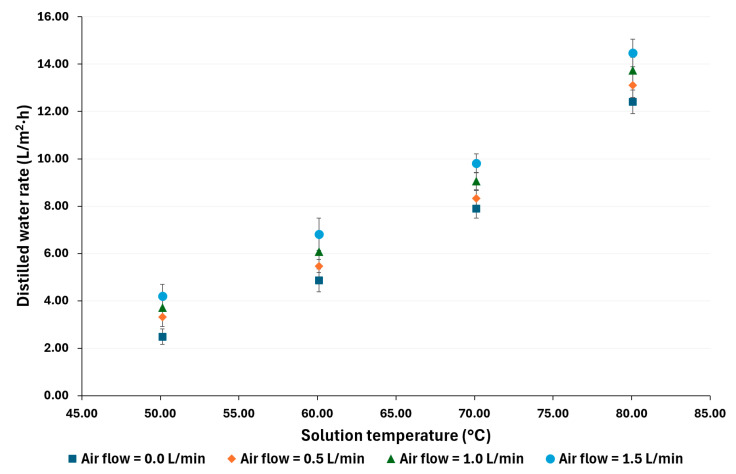
Experimental distilled water rate for a salt concentration of 70,000 ppm.

Figure 4 and Figure 5 show the distilled water rate for each experimental test operating condition. With a salt concentration of 100,000 ppm, the distilled water rate with a volumetric airflow (*V_G_*) = 0 was from 2.29 to 11.54 L/m^2^·h, with *V_G_* = 0.5 was 3.08 to 12.27 L/m^2^·h, with *V_G_* = 1.0 was 3.75 to 12.64 L/m^2^·h, and with *V_G_* = 1.5 was 4.52 to 14.10 L/m^2^·h. With a salt concentration of 70,000 ppm, the distilled water rate with *V_G_ =* 0 was from 2.48 to 12.40 L/m^2^·h, with *V_G_* = 0.5 was 3.31 to 13.11 L/m^2^·h, with *V_G_* = 1.0 was 3.70 to 13.74 L/m^2^·h, and with *V_G_* = 1.5 was 4.19 to 14.47 L/m^2^·h. When *V_G_ =* 0, the membrane module operates as an AGMD unit, and, as can be appreciated, the distilled water rate increases as the salt solution temperature increases; this behavior is consistent with what has been reported in the literature for high-concentration saline solutions [38,39]. In addition, as the airflow increases, the distilled water also increases. It is remarkable that, at low salt solution temperatures, such as 50 °C, the distilled water rate was enhanced 97% at the highest air flow value compared to the rate without air injection. On the other hand, the enhancement in the water distilled rate decreased as the solution temperature increased; for example, at 80 °C, the distilled water rate was enhanced by 22%. Ibarra-Bahena et al. [37] reported that, for high-concentration saline solutions, the mass transfer resistance decreases as the temperature increases. Thus, the effect of the improved flow characteristics is lower than at lower operating temperatures. In addition, this behavior was observed in previous studies, which suggests that gas injection at low-temperature feed solution flow could improve the distilled flux more effectively than at higher feed solution temperatures [16].

With a salt concentration of 70,000 ppm, the highest distilled water rate enhancement was 69%, with the highest air flow and the lowest operation temperature, while the highest operation temperature was 17%. The maximum distilled water rate was 14.47 L/m^2^·h at 80 °C with an airflow of 1.5 L/min and the lowest salt concentration, while the minimum value was 2.29 L/m^2^·h at 50 °C without air injection and the highest salt concentration.

**Figure 6:**
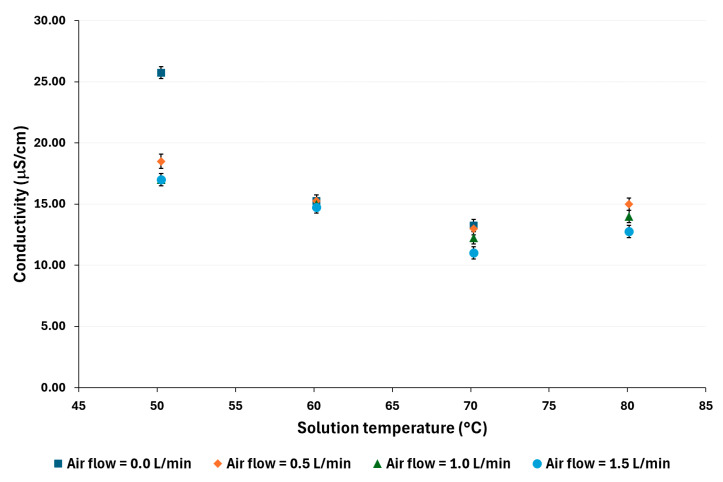
Distilled water conductivity for a salt concentration of 100,000 ppm.

**Figure 7:**
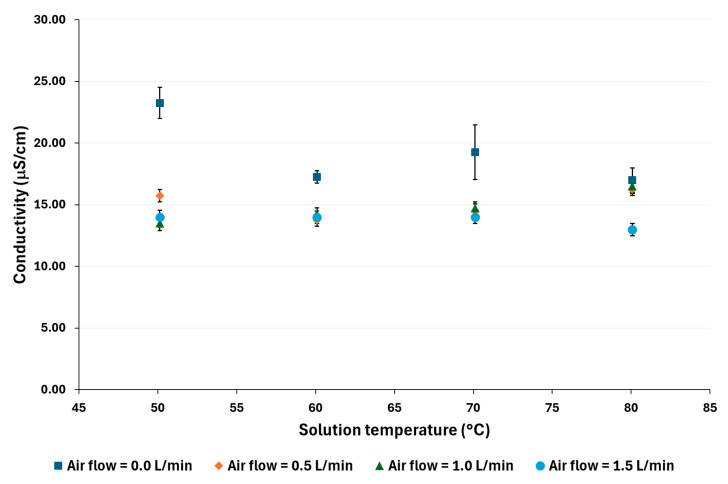
Distilled water conductivity for a salt concentration of 70,000 ppm.

Figure 6 and Figure 7 show that the distilled water conductivity decreases as the air flow increases, particularly at low solution temperatures. For example, at 50 °C and 100,000 ppm, a reduction of 66% was observed, while at the same temperature and 70,000 ppm, the reduction was 60%. On the other hand, at 80 °C, the reductions were 17% and 30% for 100,000 ppm and 70,000 ppm, respectively.

A comparison of the experimental distilled water rates reported in the literature for gas-injection MD configurations is presented in Table 3. The results are in accordance with the reported performance of the different MD systems, where the VMD configuration achieves a higher distilled water rate compared to the DCMD and AGMD configurations [40]. In addition, it is notable that there is a wide knowledge gap in analyzing the gas-injection for AGMD configuration.

### 4.2. Model Validation

**Figure 8:**
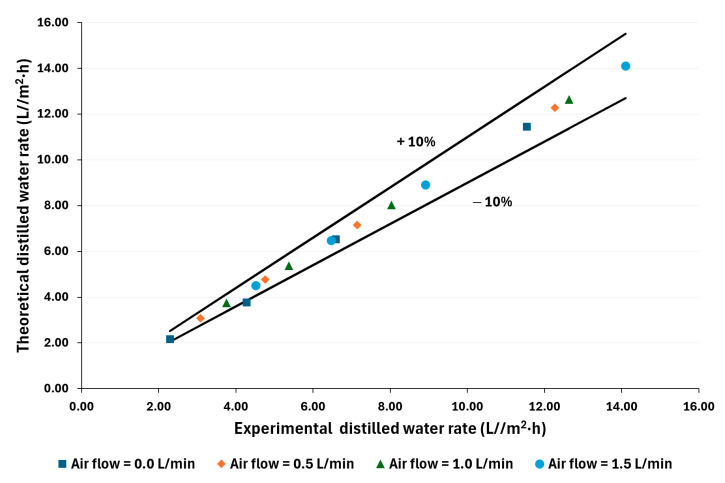
Comparison between theoretical and experimental distilled water rates for 100,000 ppm.

**Figure 9:**
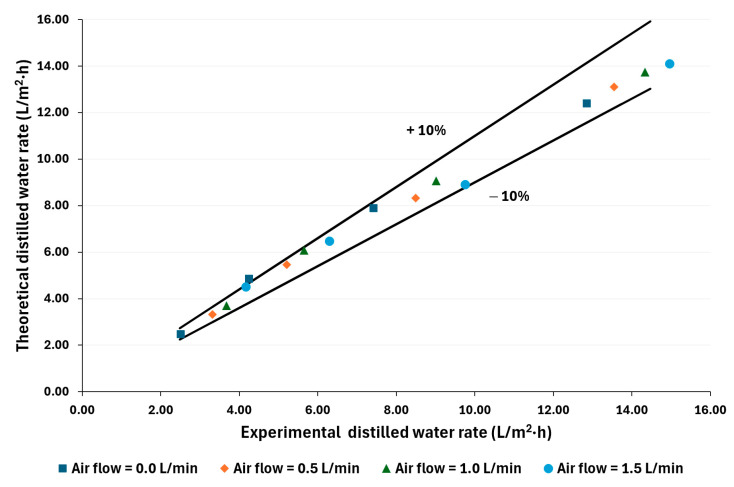
Comparison between theoretical and experimental distilled water rates for 70,000 ppm.

The distilled water rate was calculated based on the one-dimensional heat and mass transfer model. Figure 8 and Figure 9 compare theoretical and experimental values. As can be seen, 94% of the calculated values fall within ±10% of the experimental data for both salt concentration conditions. This validation provides a mathematical approach to improving the AGMD systems with air injection.

## 5. Conclusions

An air-injection-AGMD module was described and performed. Thirty-two operating conditions were tested. The effect of the saline solution temperature, salt concentration, and air flow on the distilled water rate were evaluated. According to the results, as the solution temperature and the air flow increase, the distilled water rate also increases. However, the distilled water rate was enhanced by 97% at the lowest tested temperature and the highest air flow, at a concentration of 100,000 ppm.

On the other hand, at the highest solution temperature, the distilled water was enhanced by 22% at the highest air flow rate compared to the conventional condition (without air injection) at the same salt concentration. With a salt concentration of 70,000 ppm, the highest enhancement in distilled water rate was 69% at the highest air flow and the lowest operation temperature, while at the highest operation temperature, it was 17%. The highest distilled water rate, with a salt concentration of 100,000 ppm, was 14.10 L/m^2^·h at 80 °C and *V_G_ =* 1.5 L/min. With a salt concentration of 70,000, the rate was 14.47 L/m^2^·h under the same operating conditions.

According to the experimental results, distilled water quality improved with the air injection, as a conductivity reduction of 66% and 60% was observed at salt concentrations of 100,000 ppm and 70,000 ppm, respectively, at the lowest solution temperature. Compared with other MD configurations which use gas injection, there is still room for improvement in analyzing different operation conditions for AMGD.

Using the mathematical model, 94% of the calculated values fall within ±10% of the experimental data for both salt concentration conditions. Air injection can improve the AGMD systems, particularly those driven by low-temperature sources.

## Figures and Tables

**Table 1:** Uncertainty of the measured variables.

Variable	Sensor/Instrument	Operation Range	Uncertainty
Temperature	Type T thermocouple	−250 to 350 °C	±0.5 °C
Volumetric flow	Turbine flowmeter	0 to 30 L/min	±1.0 L/min
Air volumetric flow	Analogical flowmeter	0 to 2 L/min	±4% *
Distilled water volume	Volumetric test tube	0 to 100 mL	±1.0 mL
Conductivity	Multiparameter device	0 to 200 µS/cm	±1 µS/cm

* f.s., full scale.

**Table 2:** Experimental operating conditions.

Parameter	Value
Salt concentration (ppm)	100,000
	70,000
Cooling water volumetric flow (L/min)	13.5 ± 0.2
Salt solution flow (L/min)	4.0 ± 1.8
Salt solution temperature (°C)	80.1 ± 0.1 70.2 ± 0.1 60.2 ± 0.1 50.3 ± 0.1
Air volumetric flow (L/min)	0.0 ± 0.0
	0.5 ± 0.05
	1.0 ± 0.05
	1.5 ± 0.05
Cooling water temperature (°C)	20.1 ± 0.1

**Table 3:** Comparison of the experimental distilled water rates reported in the literature for gas-injection MD configurations.

Reference	MD Configuration	Salt Concentration (g/L)	*T_L_* (°C)	* T_Con_ (°C)	*V_L_* (L/min)	*V_G_* (L/min)	*N_w_* (kg/m^2^·h)
[12]	VMD	35 and 50	75	NA	NA	NA	15.2 to 22.6
[13]	DCMD	35	40 to 60	25	1.0 to 2.0	0.04 to 0.06	5.3 to 20.1
[14]	DCMD	35 and 50	75	NA	NA	NA	1.8 to 2.8
[16]	DCMD	35 to 87.5	40 to 60	25	1.0 to 2.0	0.002 to 0.1	4.9 to 24.3
[31]	AGMD	35	60	20	0.67	0 to 1.33	0.9 to 4.4
[41]	VMD	36.3	70	NA	NA	NA	32 to 46
This work	AGMD	70 and 100	50 to 80	20.1	4.0	0 to 1.5	2.3 to 14.5

* T_Con_ is the condensing temperature.

## Data Availability

The data presented in this study are available on request from the corresponding author.

## References

[1] Edalatpour M., Aryana K., Kianifar A., Tiwari G.N., Mahian O., Wongwises S. (2016). Solar stills: A review of the latest developments in numerical simulations. Sol. Energy.

[2] Jones E., Qadir M., van Vliet M.T.H., Smakhtin V., Kang S.-M. (2019). The state of desalination and brine production: A global outlook. Sci. Total Environ..

[3] Olabi A.G., Elsaid K., Rabaia M.K.H., Askalany A.A., Abdelkareem M.A. (2020). Waste heat-driven desalination systems: Perspective. Energy.

[4] Subramani A., Jacangelo J.G. (2015). Emerging desalination technologies for water treatment: A critical review. Water Res..

[5] Lawson K.W., Lloyd D.R. (1997). Membrane distillation. J. Membr. Sci..

[6] Alkhudhiri A., Darwish N., Hilal N. (2012). Membrane distillation: A comprehensive review. Desalination.

[7] Drioli E., Ali A., Macedonio F. (2015). Membrane distillation: Recent developments and perspectives. Desalination.

[8] Martínez-Díez L., Vázquez-González M.I. (1996). Temperature polarization in mass transport through hydrophobic porous membranes. AIChE J..

[9] Lee J.-G., Kim W.-S., Choi J.-S., Ghaffour N., Kim Y.-D. (2016). A novel multi-stage direct contact membrane distillation module: Design, experimental and theoretical approaches. Water Res..

[10] Mengual J.I., Khayet M., Godino M.P. (2004). Heat and mass transfer in vacuum membrane distillation. Int. J. Heat Mass Transf..

[11] Shim S.-M., Lee J.-G., Kim W.-S. (2014). Performance simulation of a multi-VMD desalination process including the recycle flow. Desalination.

[12] Dong C., Huang Y., Zhang L. (2023). Slug flow-enhanced vacuum membrane distillation for seawater desalination: Flux improvement and anti-fouling effect. Sep. Purif. Technol..

[13] Kim Y.-B., Lee H.-S., Gil G.-W., Ji H., Kim Y.-D. (2023). Comprehensive experimental and theoretical investigations on the effect of microbubble two-phase flow on the performance of direct-contact membrane distillation. Water Res..

[14] Dong C., Huang Y., Lin H., Zhang L. (2022). Performance intensification and anti-fouling of the two-phase flow enhanced direct contact membrane distillation for seawater desalination. Desalination.

[15] Cao G., Ma Q., Li J., Wang S., Wang C., Lu H., Zheng Y. (2021). Seawater desalination based on a bubbling and vacuum-enhanced direct contact membrane distillation. Int. J. Chem. Eng..

[16] Kim Y.B., Lee H.S., Francis L.J., Kim Y.D. (2019). Innovative swirling flow-type microbubble generator for multi-stage DCMD desalination system: Focus on the two-phase flow pattern, bubble size distribution, and its effect on MD performance. J. Membr. Sci..

[17] Wibisono Y., Cornelissen E.R., Kemperman A.J.B., van der Meer W.G.J., Nijmeijer K. (2014). Two-phase flow in membrane processes: A technology with a future. J. Membr. Sci..

[18] Tian R., Gao H., Yang X.H., Yan S.Y., Li S. (2014). A new enhancement technique on air gap membrane distillation. Desalination.

[19] Francis L., Ahmed F.E., Hilal N. (2022). Advances in Membrane Distillation Module Configurations. Membranes.

[20] Juybari H.F., Karimi M., Srivastava R., Swaminathan J., Warsinger D.M. (2023). Superhydrophobic composite asymmetric electrospun membrane for sustainable vacuum assisted air gap membrane distillation. Desalination.

[21] Omar N.M.A., Othman M.H.D., Tai Z.S., Kurniawan T.A., Puteh M.H., Jaafar J., Rahman M.A., Bakar S.A., Abdullah H. (2024). A review of superhydrophobic and omniphobic membranes as innovative solutions for enhancing water desalination performance through membrane distillation. Surf. Interfaces.

[22] Khalifa A.E., Alawad S.M., Antar M.A. (2017). Parallel and series multistage air gap membrane distillation. Desalination.

[23] Pangarkar B.L., Deshmukh S.K. (2015). Theoretical and experimental analysis of multi-effect air gap membrane distillation process (ME-AGMD). J. Environ. Chem. Eng..

[24] Zhang S., Lu X., Liu Z., Ma R., Ren K., Gu J. (2023). A novel braided structure with enhanced feed flow state for hollow fiber air-gap membrane distillation performance improvement. Desalination.

[25] Ho C.-D., Chen L., Yang Y.-L., Chen S.-T., Lim J.W., Chen Z.-Z. (2023). Permeate flux enhancement in air gap membrane distillation modules with inserting Λ-ribs carbon-fiber open slots. Membranes.

[26] Ahmed F.E., Lalia B.S., Hashaikeh R., Hilal N. (2022). Intermittent direct joule heating of membrane surface for seawater desalination by air gap membrane distillation. J. Membr. Sci..

[27] Bahar R., Hawlader R., Ariff T.F. (2015). Channeled coolant plate: A new method to enhance freshwater production from an air gap membrane distillation (AGMD) desalination unit. Desalination.

[28] Wu Z., Guo F. (2023). Finned tubular air gap membrane distillation. Membranes.

[29] Ali K., Khatab M.Z., Abdelsamie M.M., Ali M.M. (2024). Computational fluid dynamic investigation on performance of air gap membrane distillation with a rotating fan. Case Stud. Chem. Environ. Eng..

[30] Lawal D., Azeem M.A., Khalifa A., Falath W., Baroud T., Antar M. (2022). Performance improvement of an air gap membrane distillation process with rotating fan. Appl. Therm. Eng..

[31] Pan Y., Shi Y., Li H., Wang W. (2022). Experimental and numerical investigations on gas injection-enhanced air gap membrane distillation for water desalination. Ind. Eng. Chem. Res..

[32] Portillo E., Ruiz de la Rosa M., Louzara G., Ruiz J.M., Marín-Guirao L., Quesada J., González J.C., Roque F., González N., Mendoza H. (2014). Assessment of the abiotic and biotic effects of sodium metabisulphite pulses discharged from desalination plant chemical treatments on seagrass (Cymodocea nodosa) habitats in the Canary Islands. Mar. Pollut. Bull..

[33] Groothuis H., Hendal W.P. (1959). Heat transfer in two-phase flow. Chem. Eng. Sci..

[34] Cai J., Guo F. (2017). Study of mass transfer coefficient in membrane desalination. Desalination.

[35] Izquierdo-Gil M.A., Garcia-Payo M.C., Fernandez-Pineda C. (1999). Air gap membrane distillation of sucrose aqueous solutions. J. Membr. Sci..

[36] Dehesa-Carrasco U., Pérez-Rábago C.A., Arancibia-Bulnes C.A. (2013). Experimental evaluation and modeling of internal temper-atures in an air gap membrane distillation unit. Desalination.

[37] Ibarra-Bahena J., Dehesa-Carrasco U., Romero R.J., Rivas-Herrera B., Rivera W. (2017). Experimental assessment of a hydrophobic membrane-based desorber/condenser with H_2_O/LiBr mixture for absorption systems. Exp. Therm. Fluid Sci..

[38] Alkhudhiri A., Darwish N., Hilal N. (2012). Treatment of high salinity solutions: Application of air gap membrane distillation. Desalination.

[39] Alkhudhiri A., Hilal N. (2017). Air gap membrane distillation: A detailed study of high saline solution. Desalination.

[40] Ali A., Shirazi M.M.A., Nthunya L., Castro-Muñoz R., Ismail N., Tavajohi N., Zaragoza G., Quist-Jensen C.A. (2024). Progress in module design for membrane distillation. Desalination.

[41] Wu C., Li Z., Zhang J., Jia Y., Gao Q., Lu X. (2015). Study on the heat and mass transfer in air-bubbling enhanced vacuum membrane distillation. Desalination.

